# Supplementary figures and images for: Retrotransposon-based genetic variation of Poa annua populations from contrasting climate conditions
Source: PeerJ. 2019 May 15;7:e6888. doi: 10.7717/peerj.6888 (PMC6525586; doi:10.7717/peerj.6888)

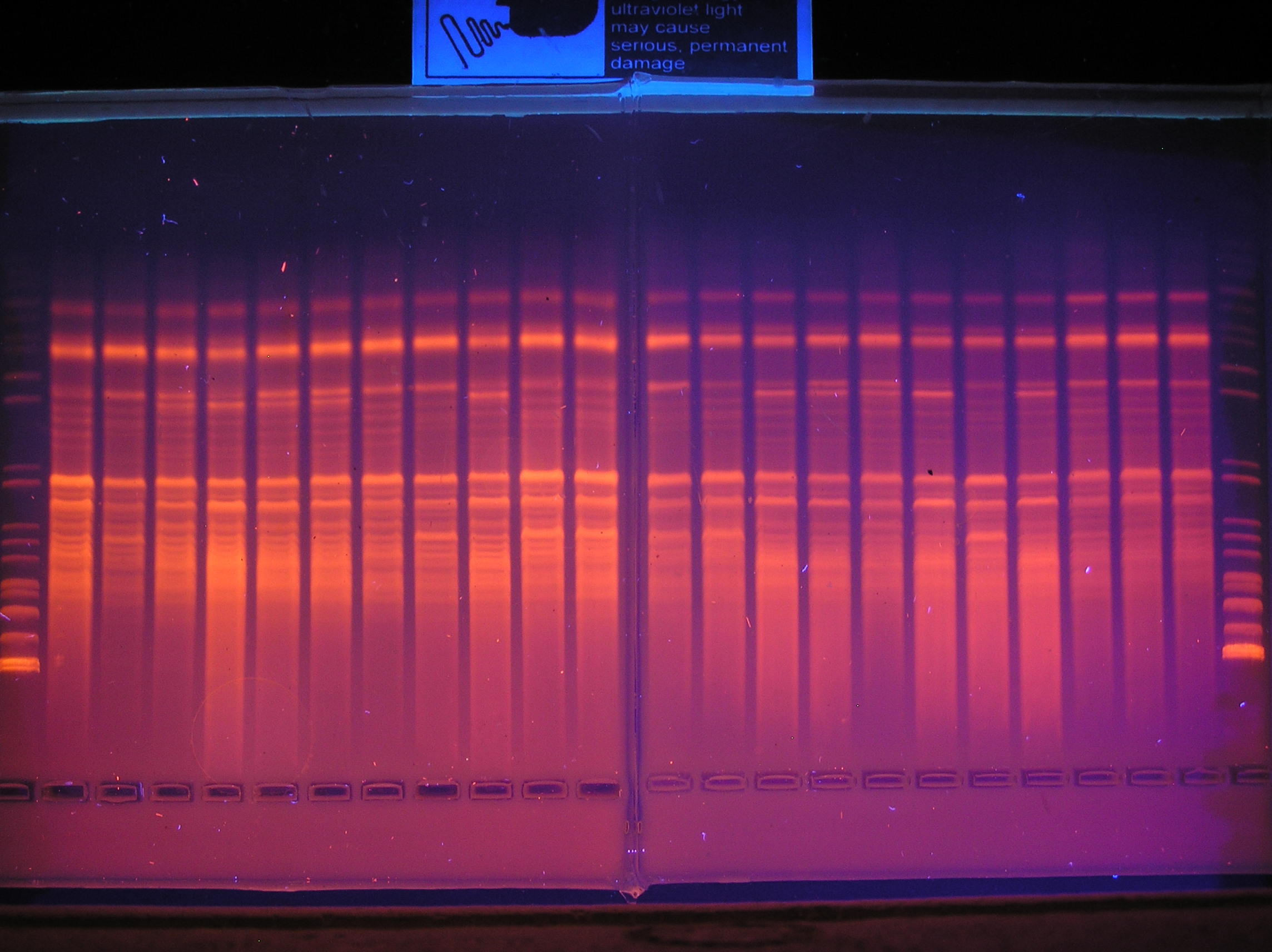

Supplement: Supplemental Information 2 [file peerj-07-6888-s002.zip › iPBS2253/2253_a.JPG]

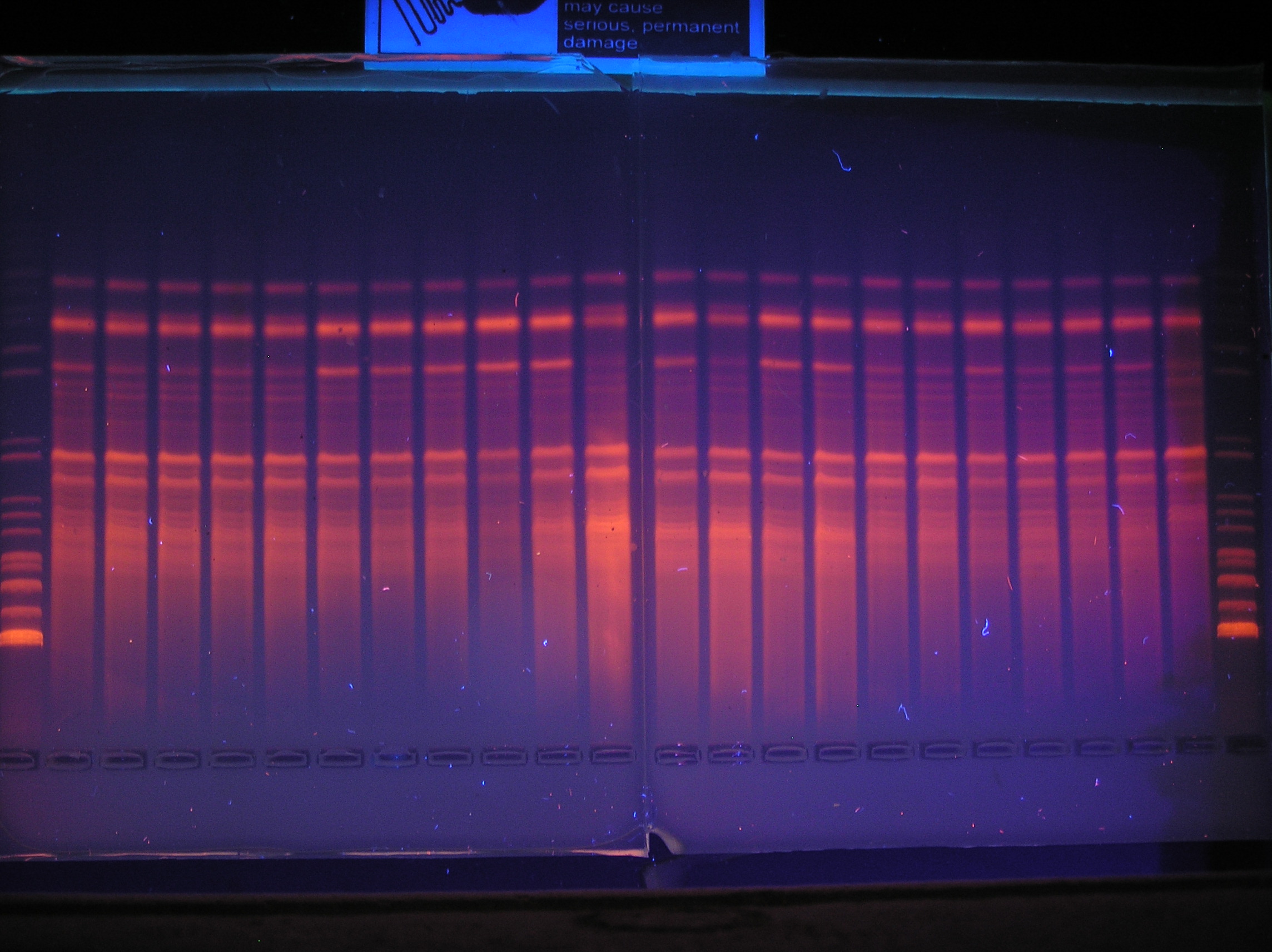

Supplement: Supplemental Information 2 [file peerj-07-6888-s002.zip › iPBS2253/2253_b.JPG]

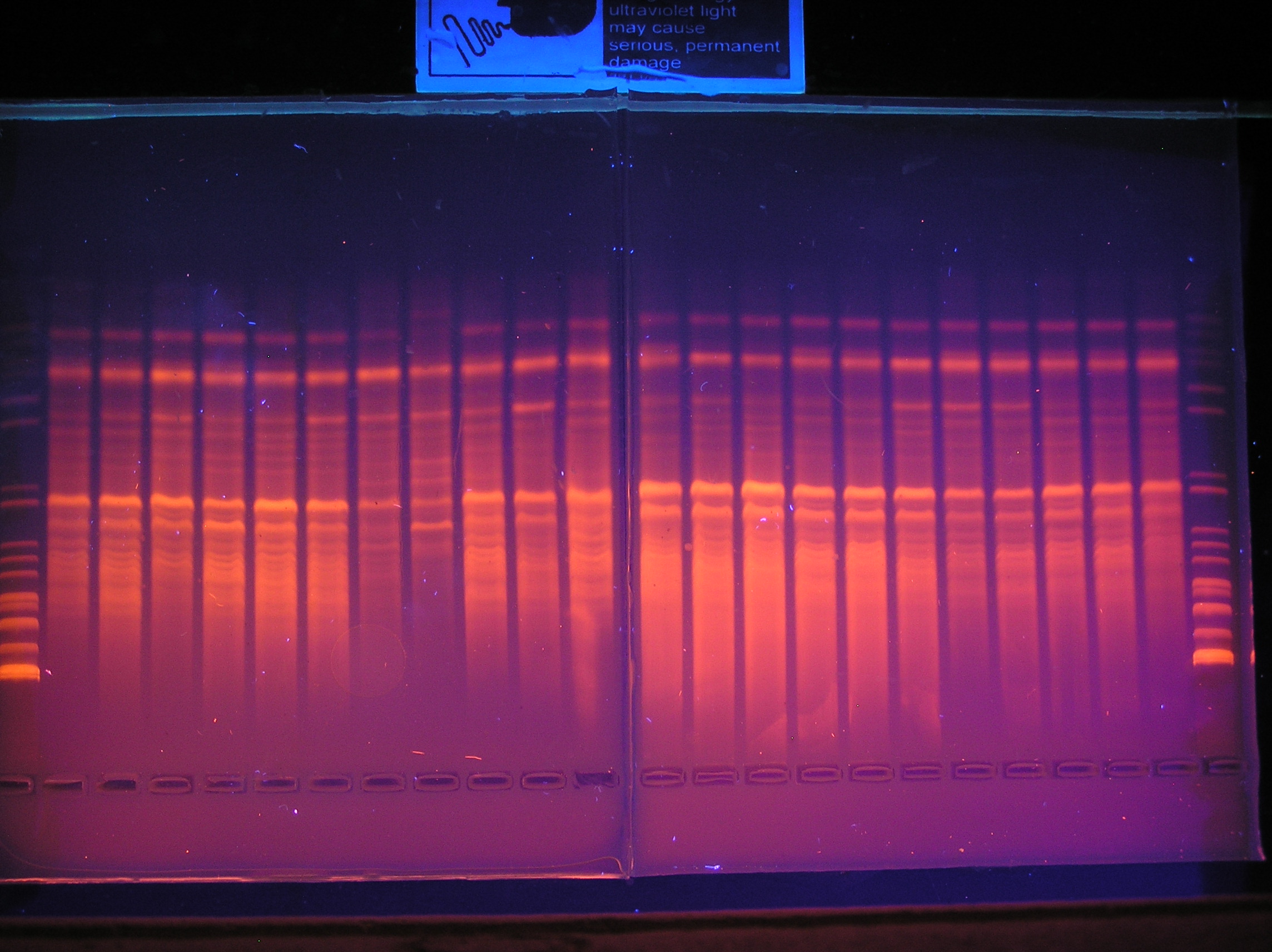

Supplement: Supplemental Information 2 [file peerj-07-6888-s002.zip › iPBS2253/2253_c.JPG]

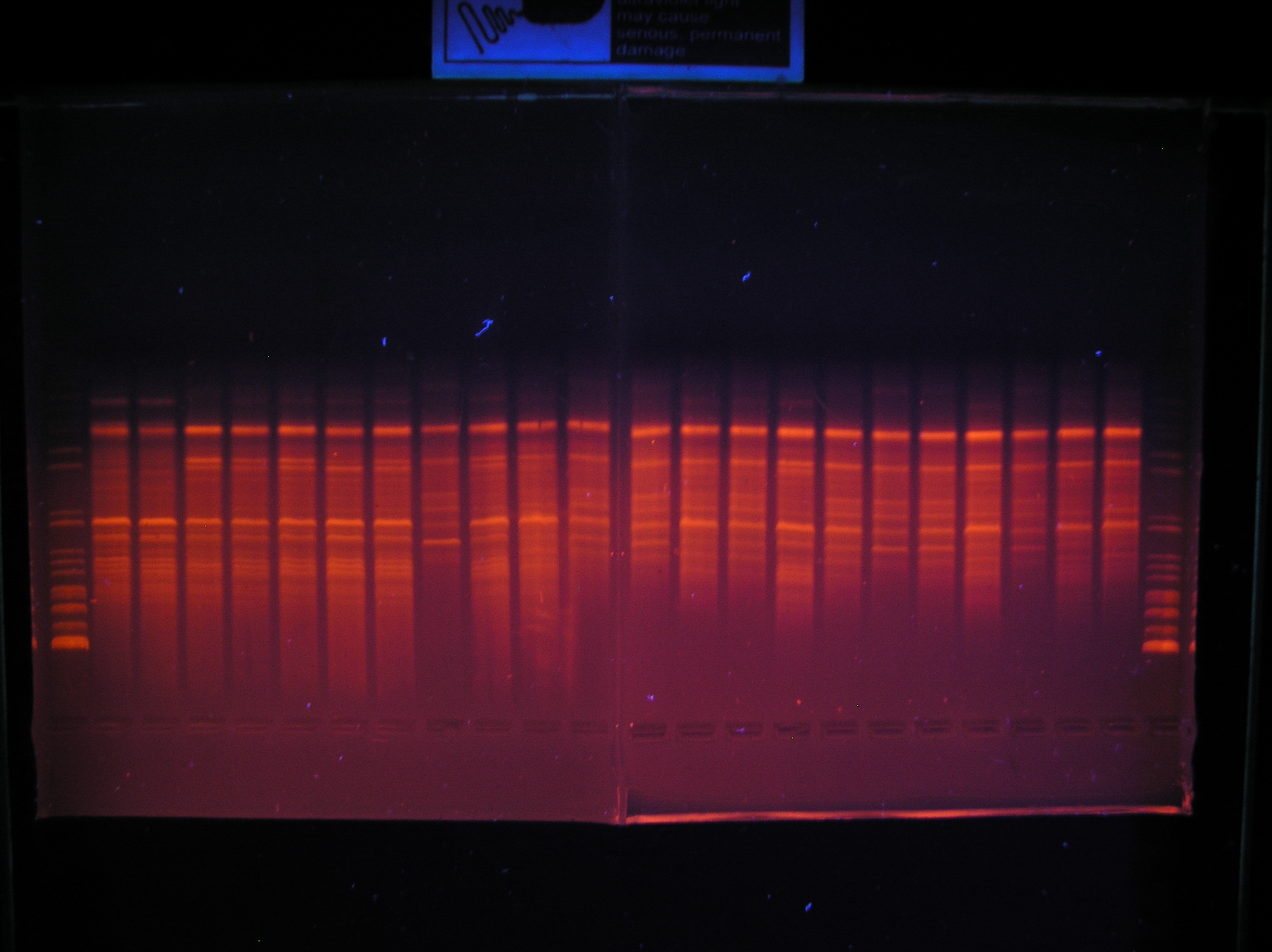

Supplement: Supplemental Information 2 [file peerj-07-6888-s002.zip › iPBS2253/2253_d.JPG]

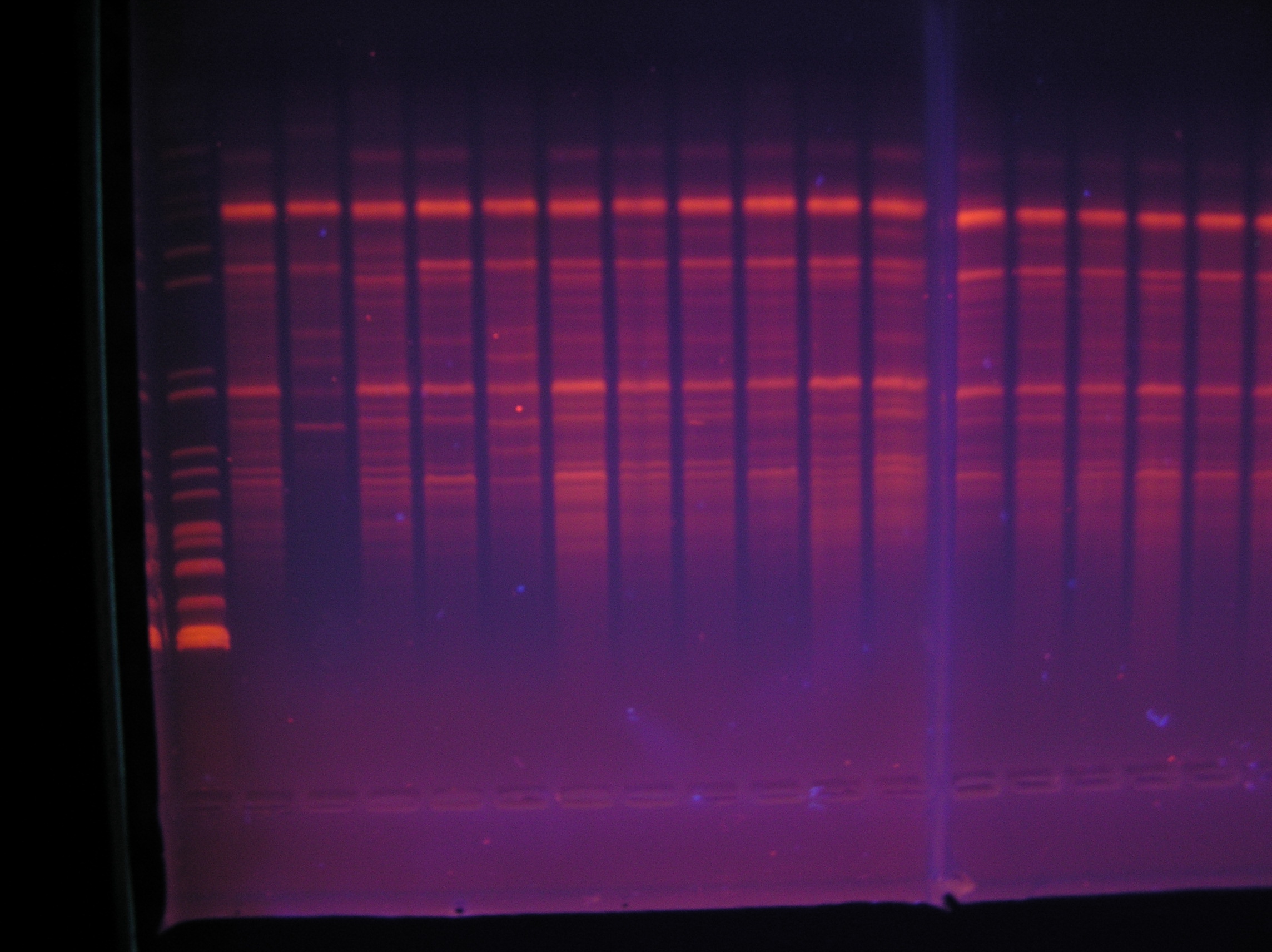

Supplement: Supplemental Information 2 [file peerj-07-6888-s002.zip › iPBS2253/2253_e1.JPG]

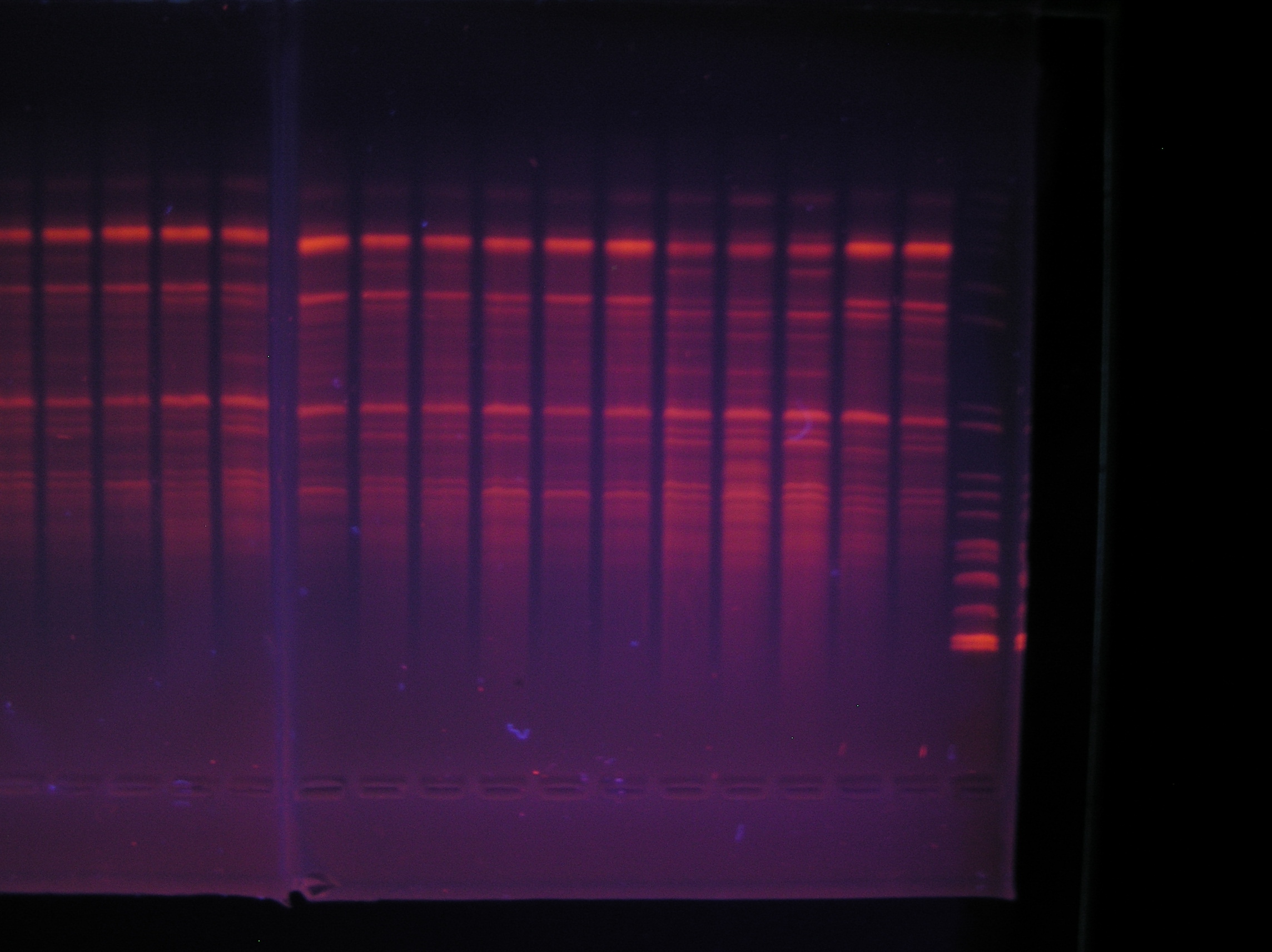

Supplement: Supplemental Information 2 [file peerj-07-6888-s002.zip › iPBS2253/2253_e2.JPG]

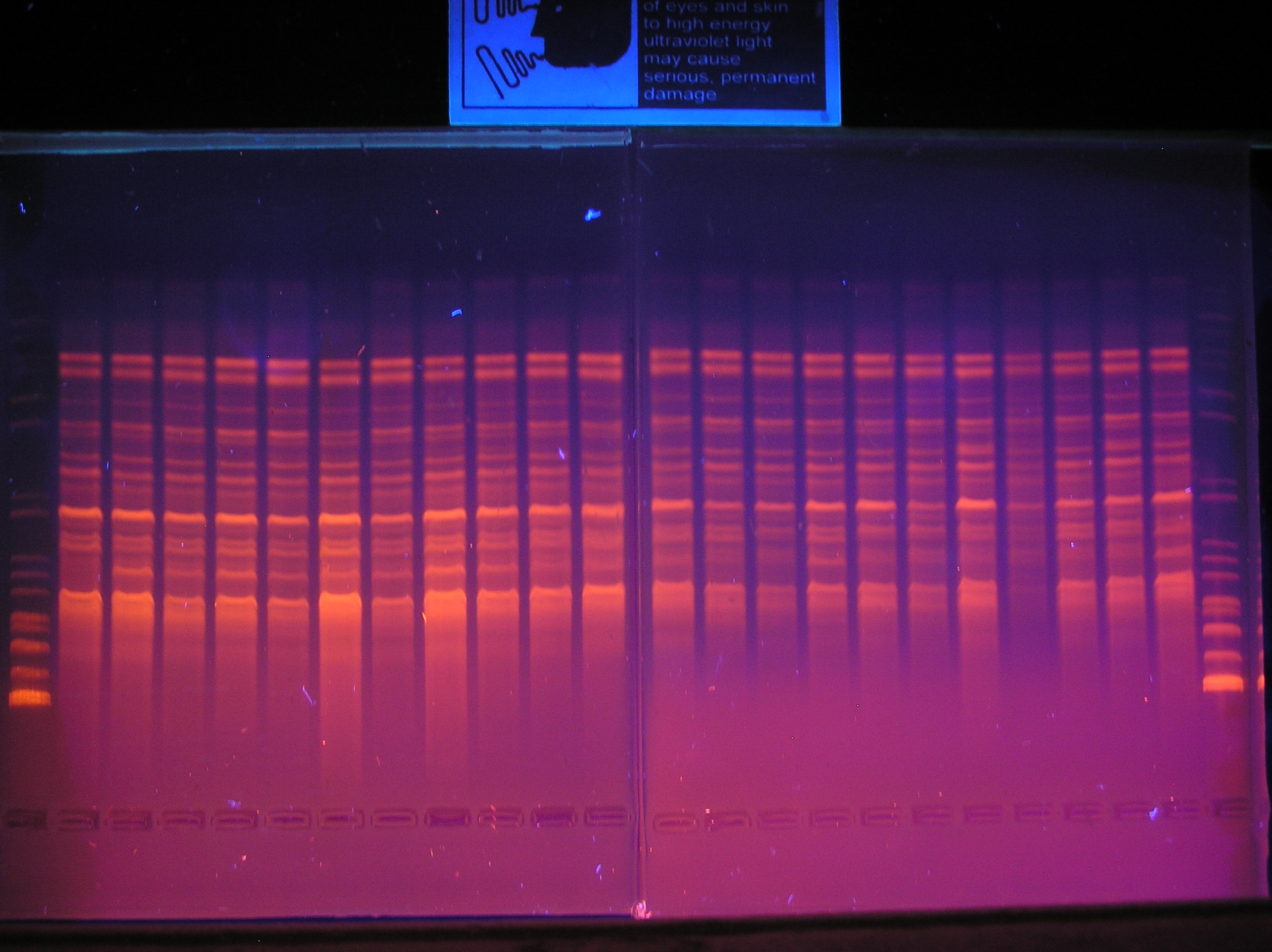

Supplement: Supplemental Information 2 [file peerj-07-6888-s002.zip › iPBS2253/2253_f.JPG]

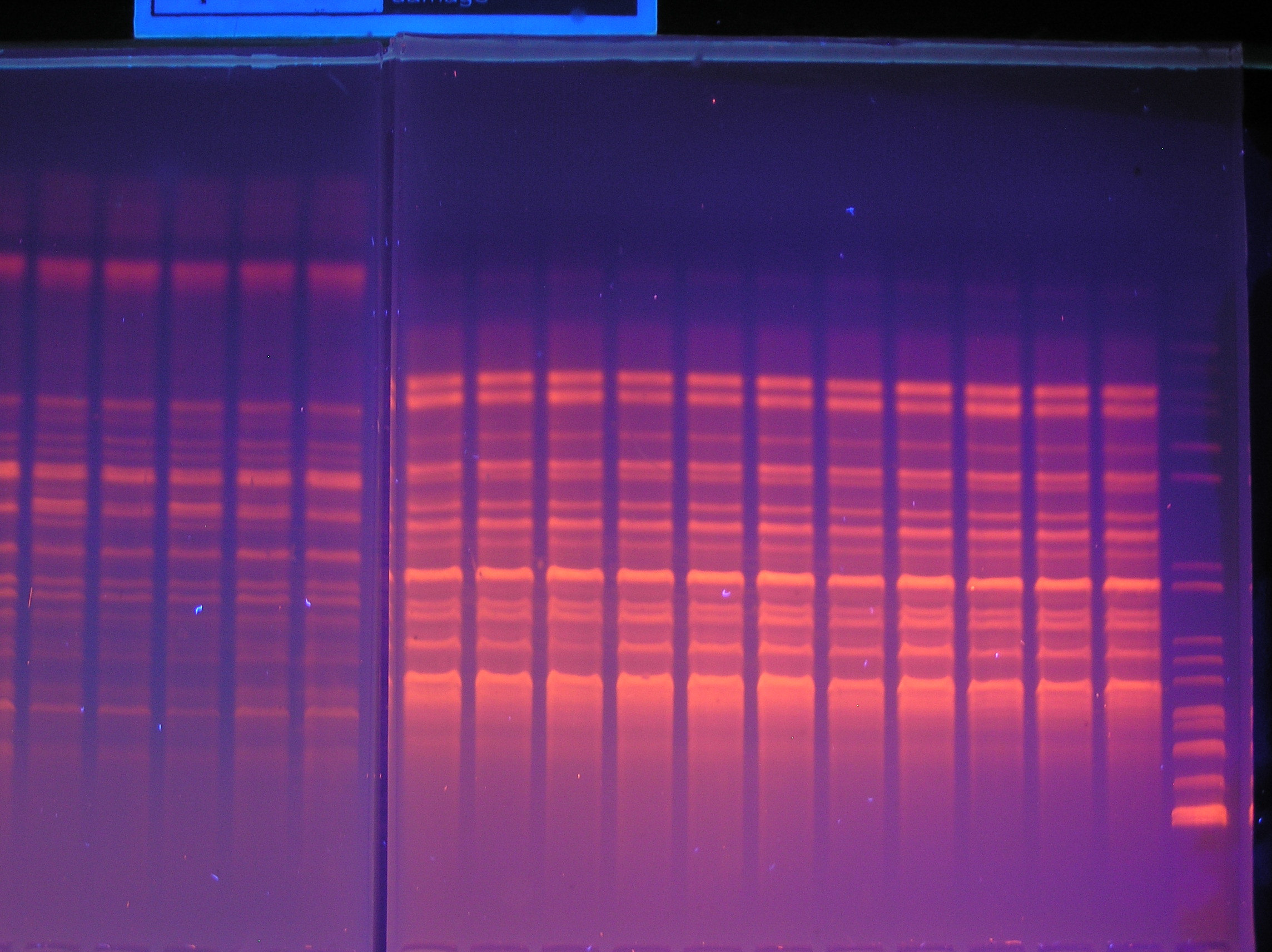

Supplement: Supplemental Information 2 [file peerj-07-6888-s002.zip › iPBS2253/2253_g.JPG]

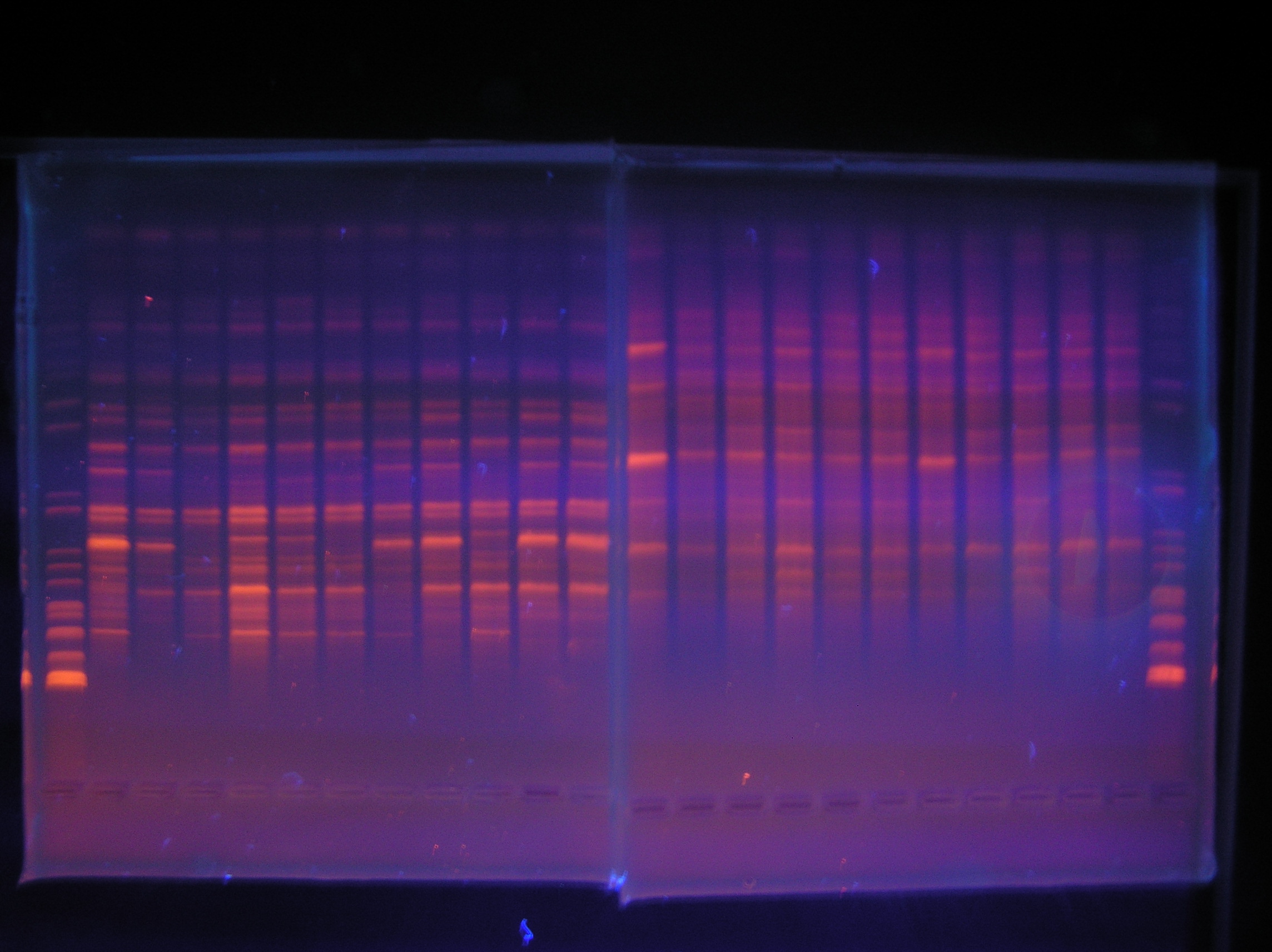

Supplement: Supplemental Information 2 [file peerj-07-6888-s002.zip › iPBS2229/2229_a1_photo_on the left.JPG]

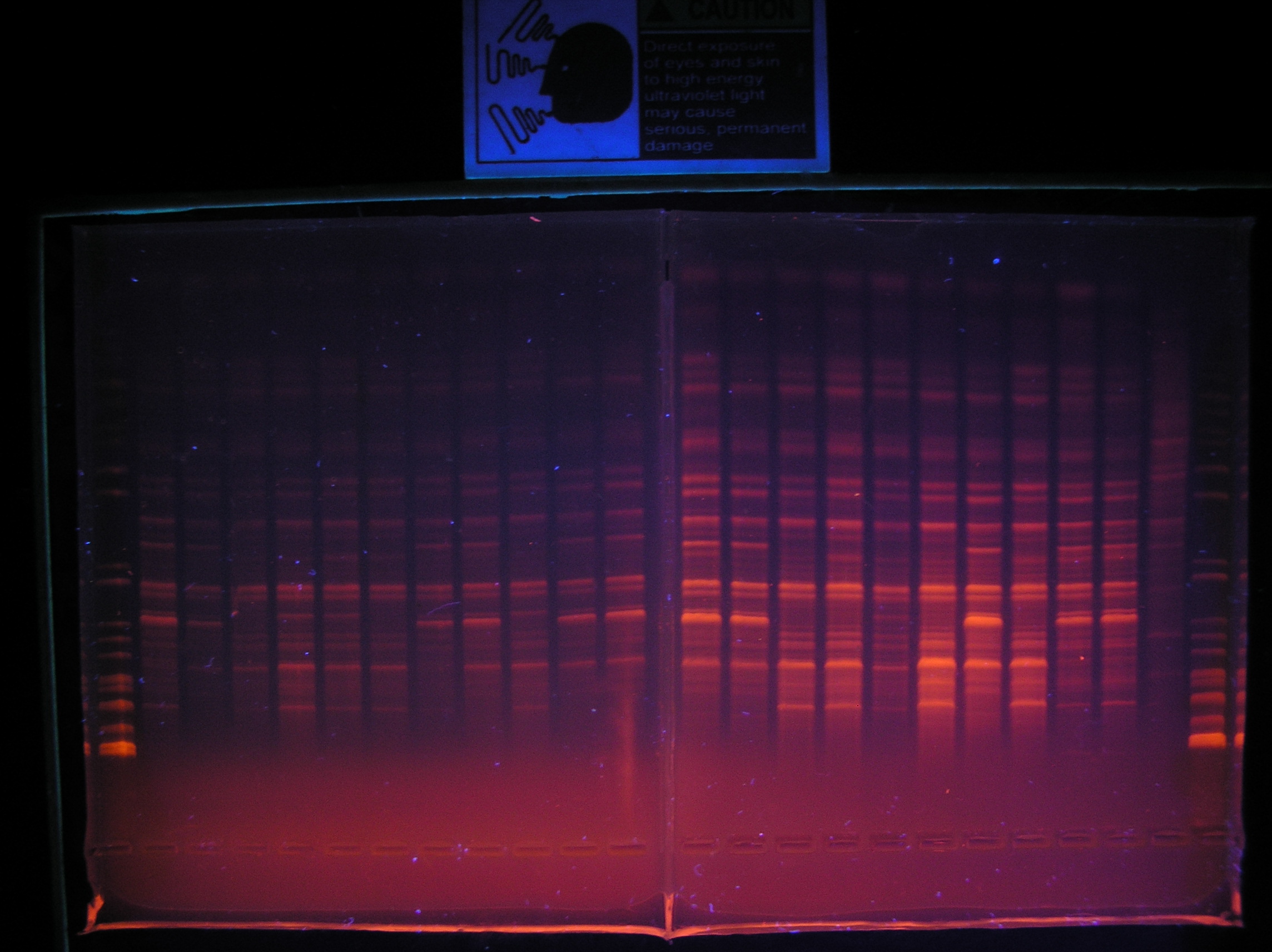

Supplement: Supplemental Information 2 [file peerj-07-6888-s002.zip › iPBS2229/2229_a2_photo on the right.JPG]

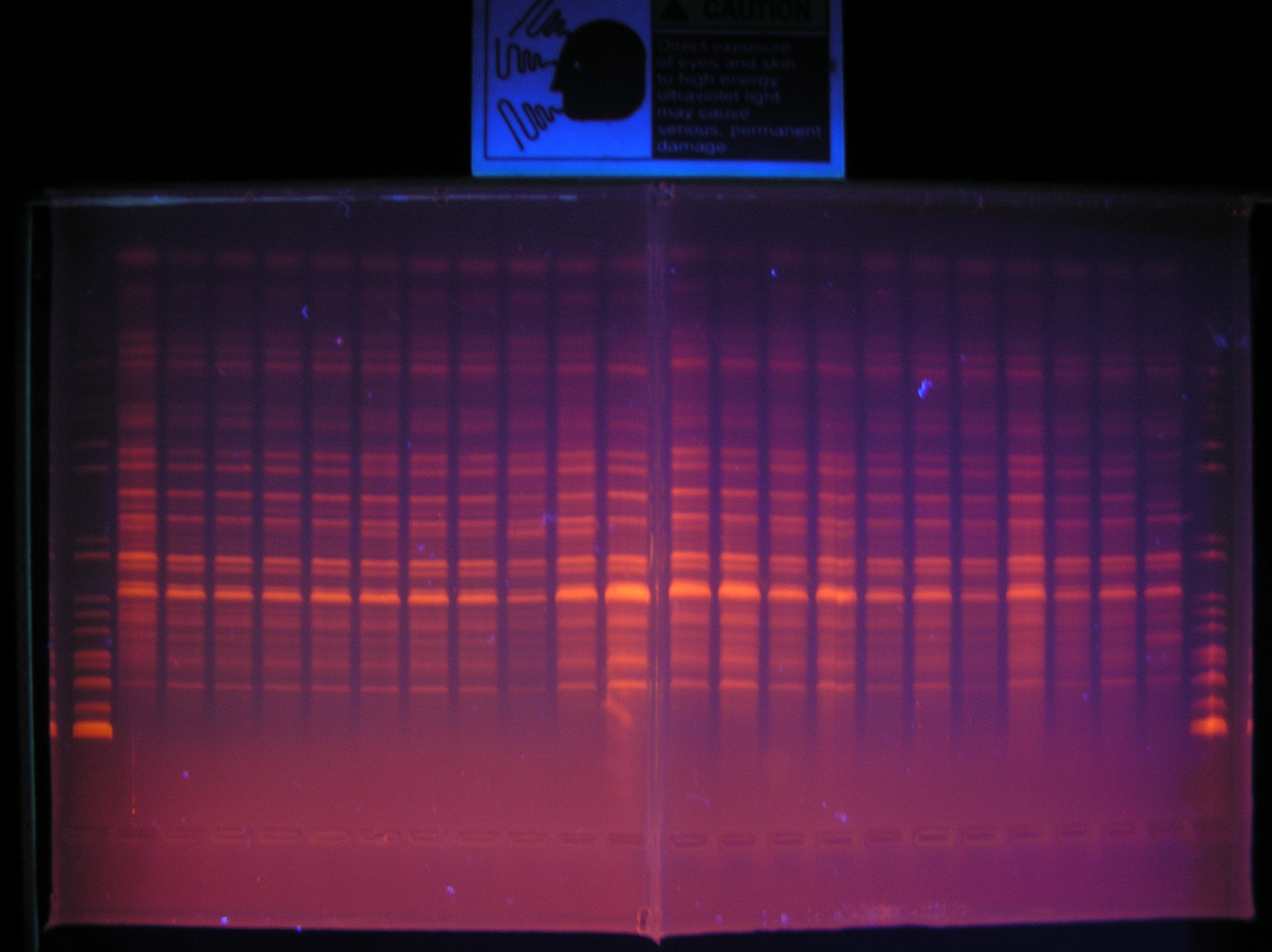

Supplement: Supplemental Information 2 [file peerj-07-6888-s002.zip › iPBS2229/2229_b.JPG]

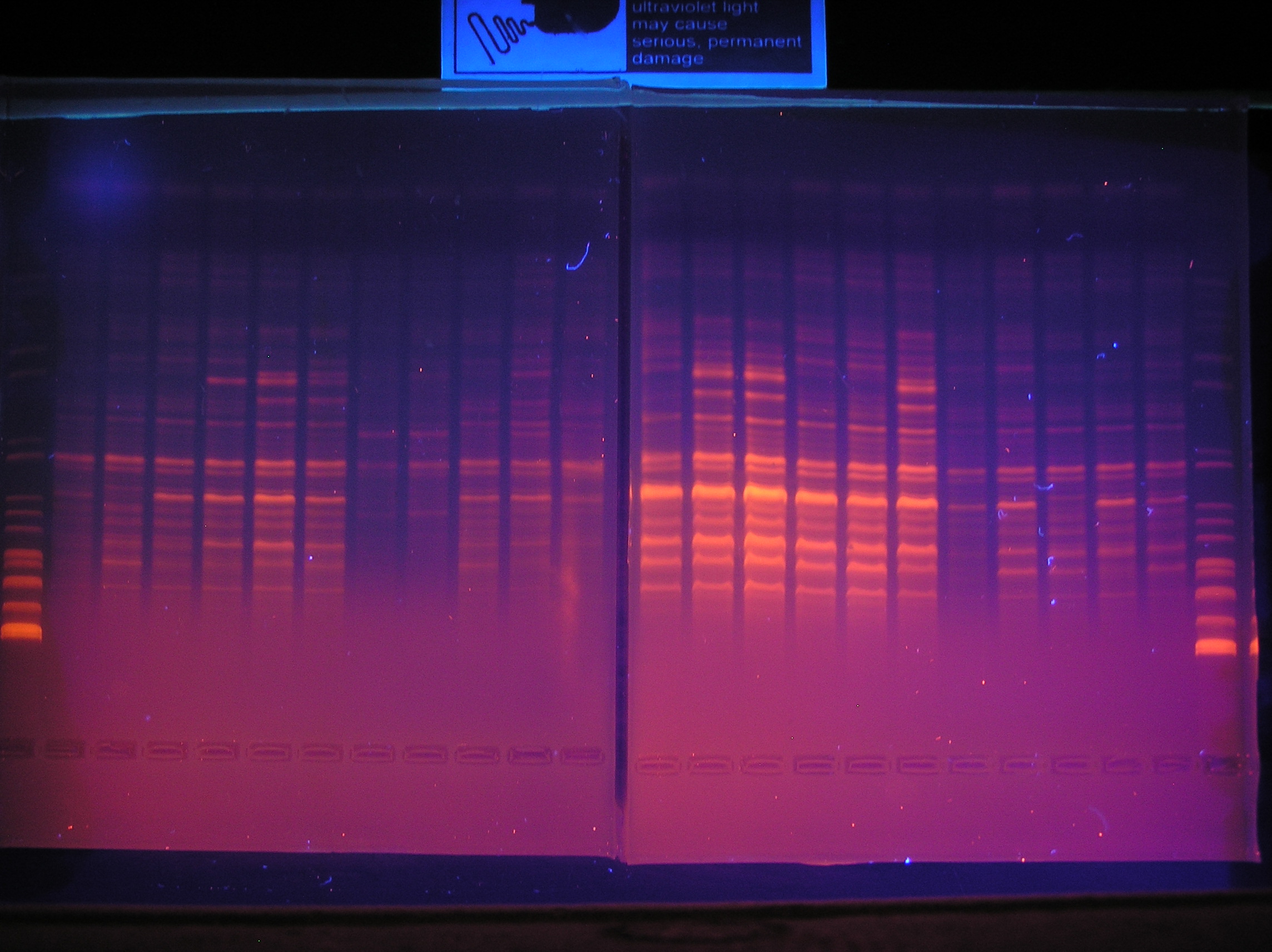

Supplement: Supplemental Information 2 [file peerj-07-6888-s002.zip › iPBS2229/2229_c.JPG]

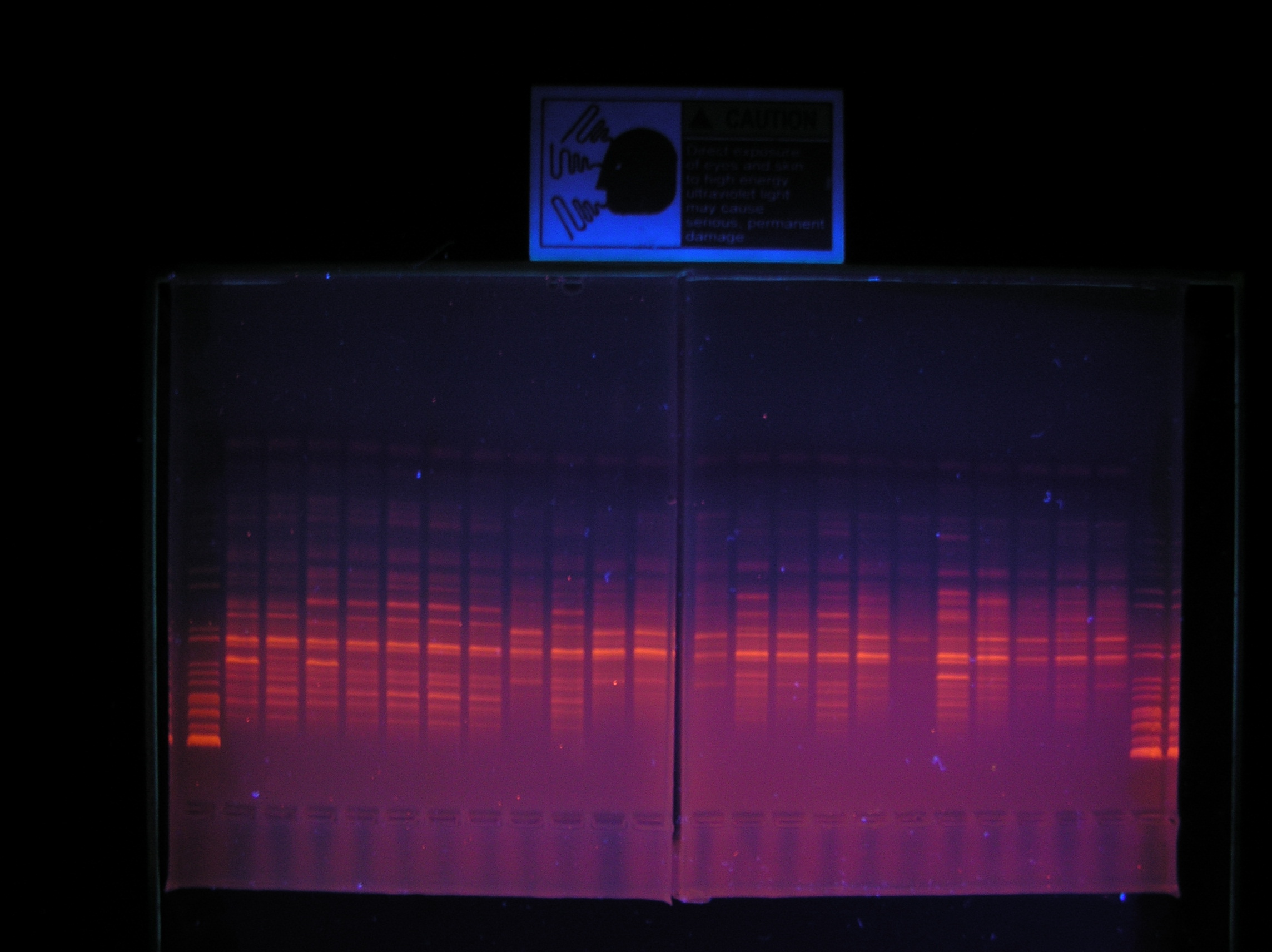

Supplement: Supplemental Information 2 [file peerj-07-6888-s002.zip › iPBS2229/2229_d.JPG]

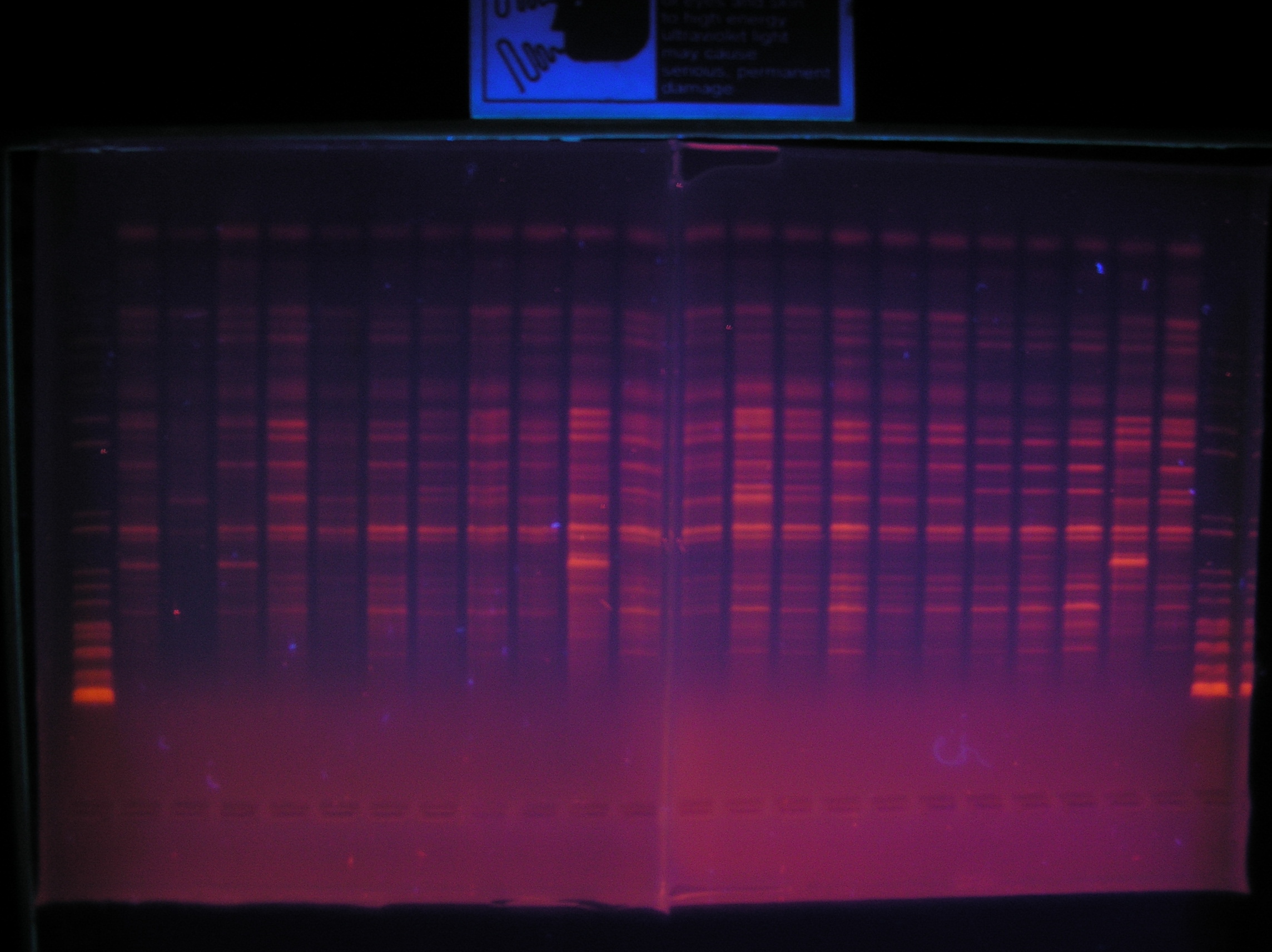

Supplement: Supplemental Information 2 [file peerj-07-6888-s002.zip › iPBS2229/2229_e.JPG]

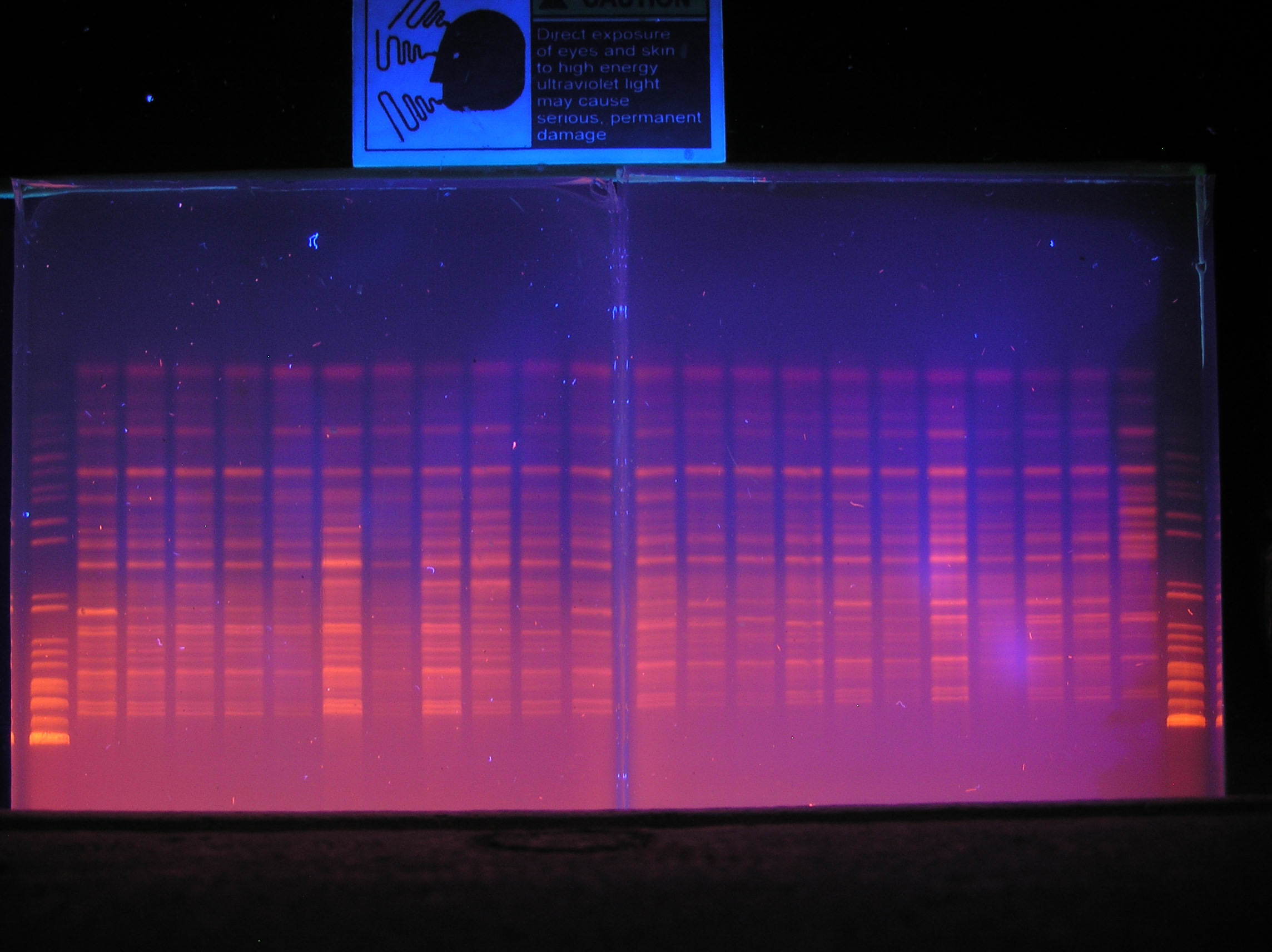

Supplement: Supplemental Information 2 [file peerj-07-6888-s002.zip › iPBS2229/2229_f.JPG]

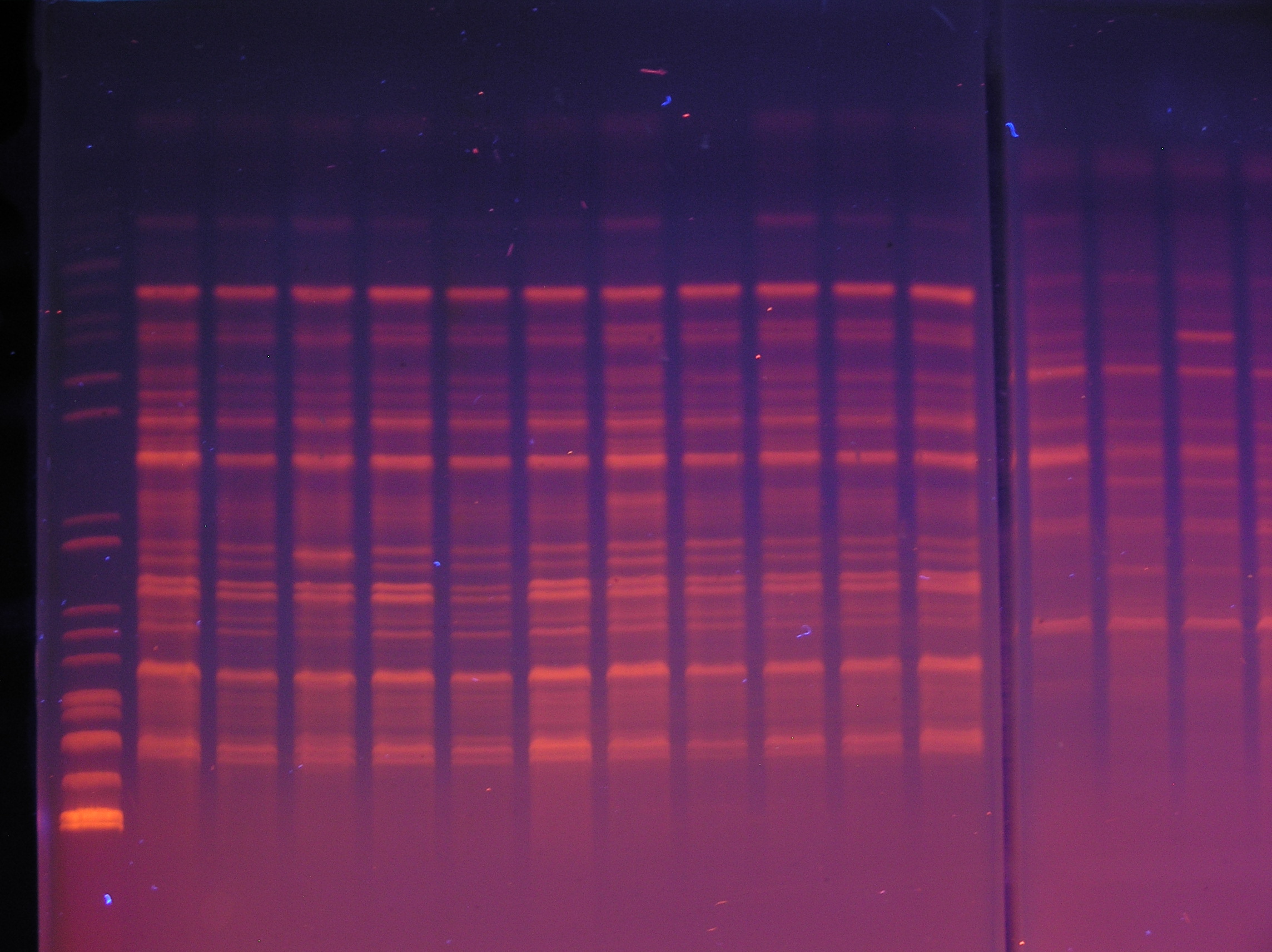

Supplement: Supplemental Information 2 [file peerj-07-6888-s002.zip › iPBS2229/2229_g.JPG]

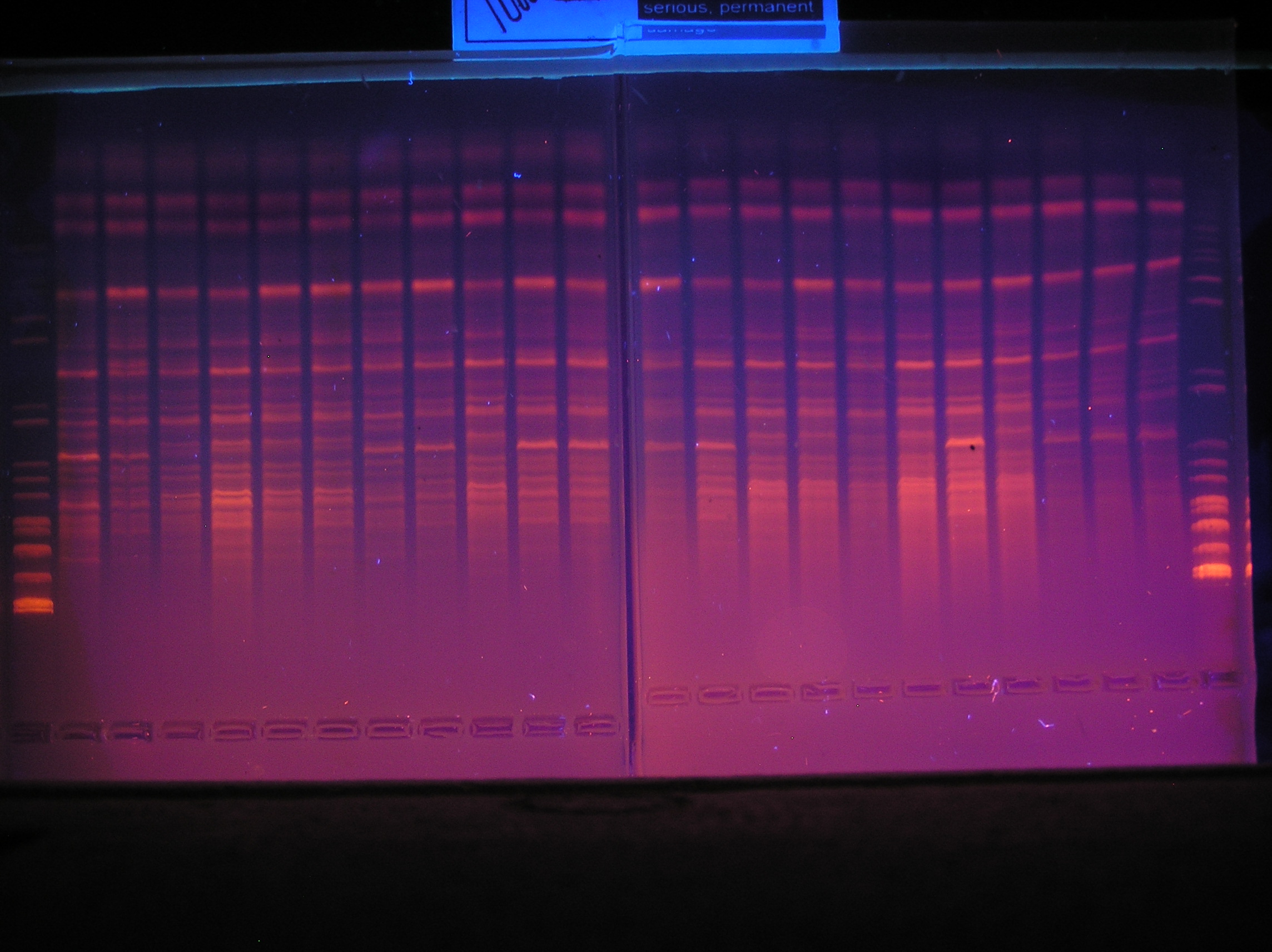

Supplement: Supplemental Information 2 [file peerj-07-6888-s002.zip › iPBS2249/2249_a.JPG]

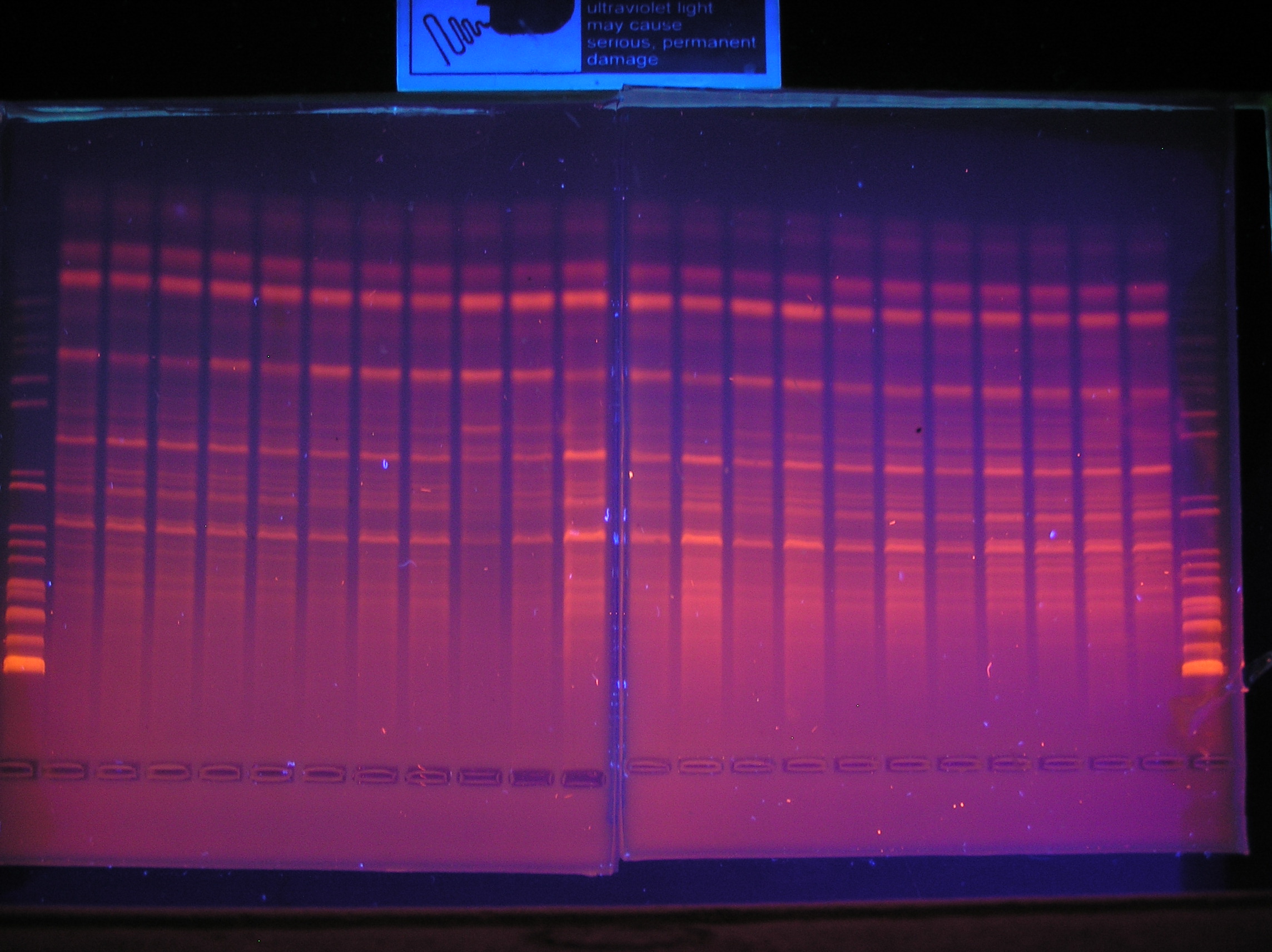

Supplement: Supplemental Information 2 [file peerj-07-6888-s002.zip › iPBS2249/2249_b.JPG]

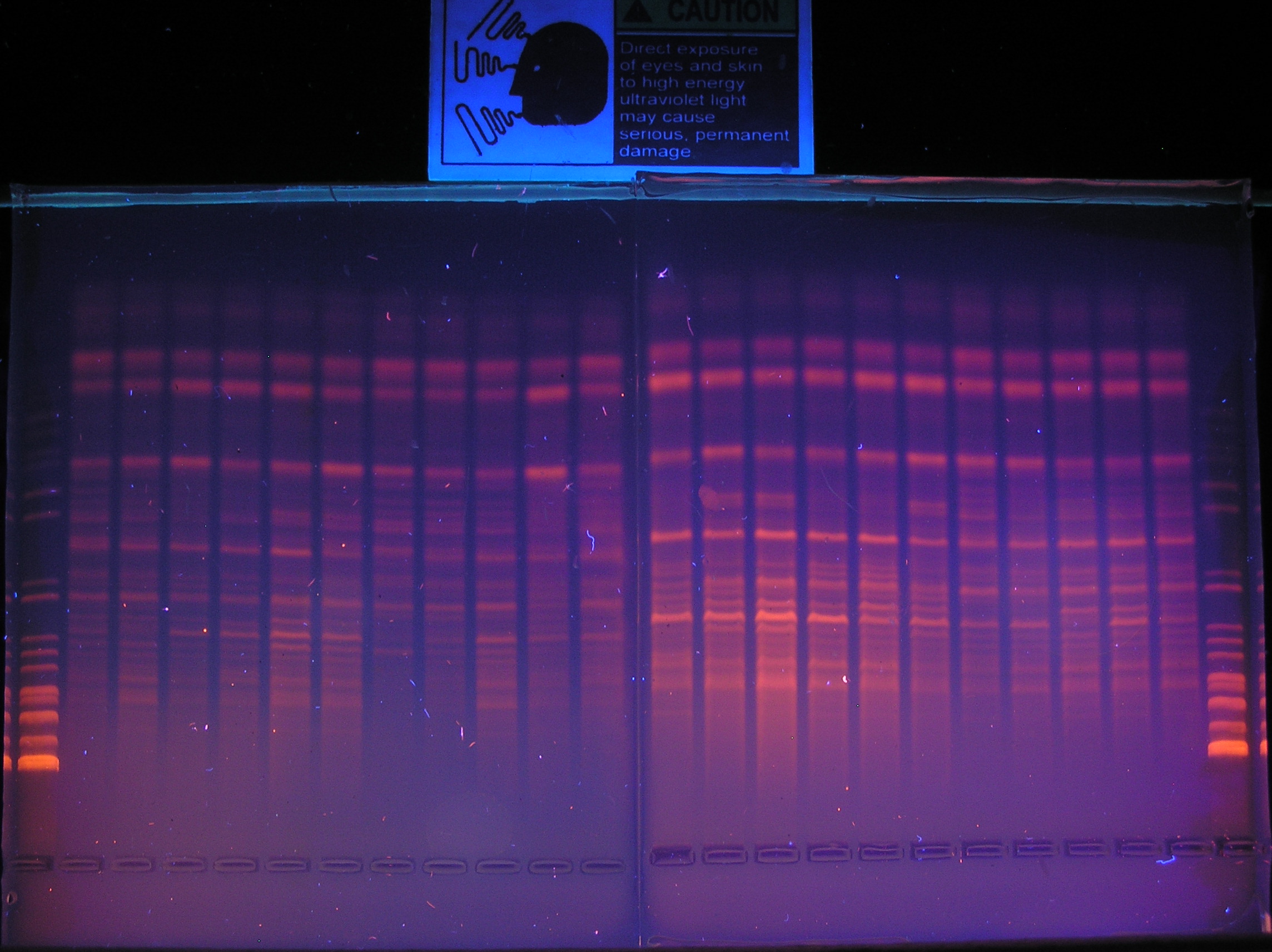

Supplement: Supplemental Information 2 [file peerj-07-6888-s002.zip › iPBS2249/2249_c.JPG]

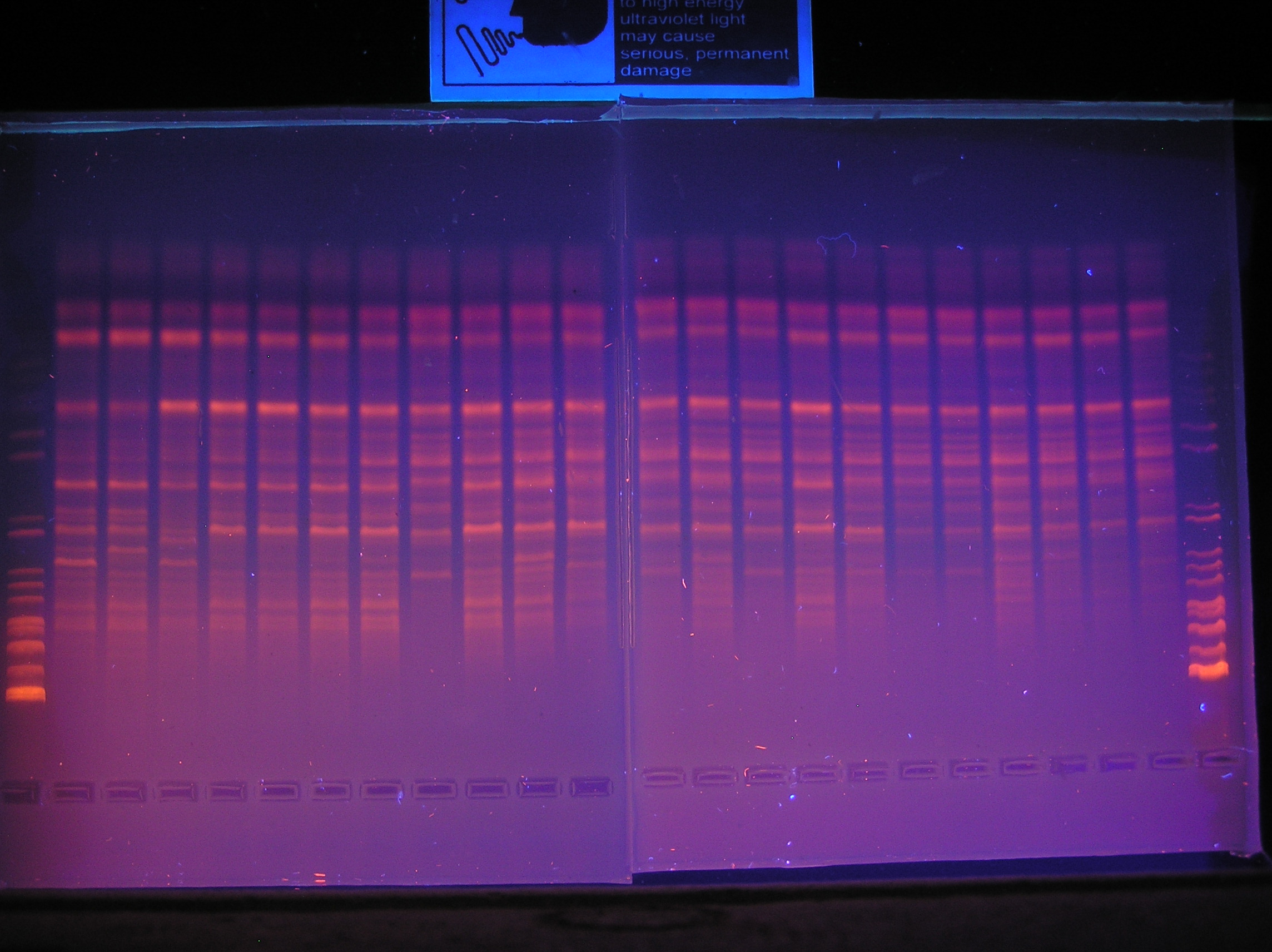

Supplement: Supplemental Information 2 [file peerj-07-6888-s002.zip › iPBS2249/2249_d.JPG]

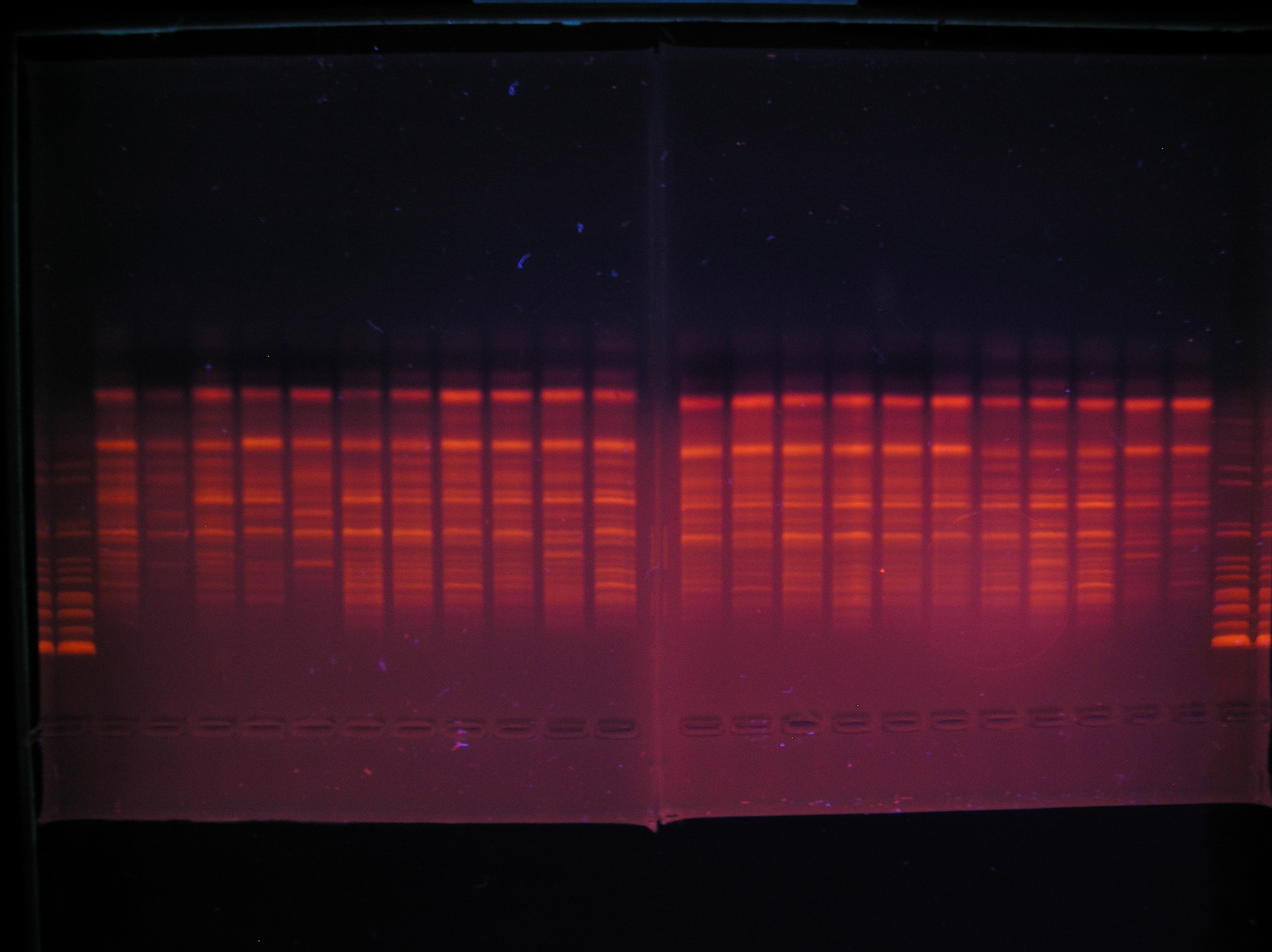

Supplement: Supplemental Information 2 [file peerj-07-6888-s002.zip › iPBS2249/2249_e.JPG]

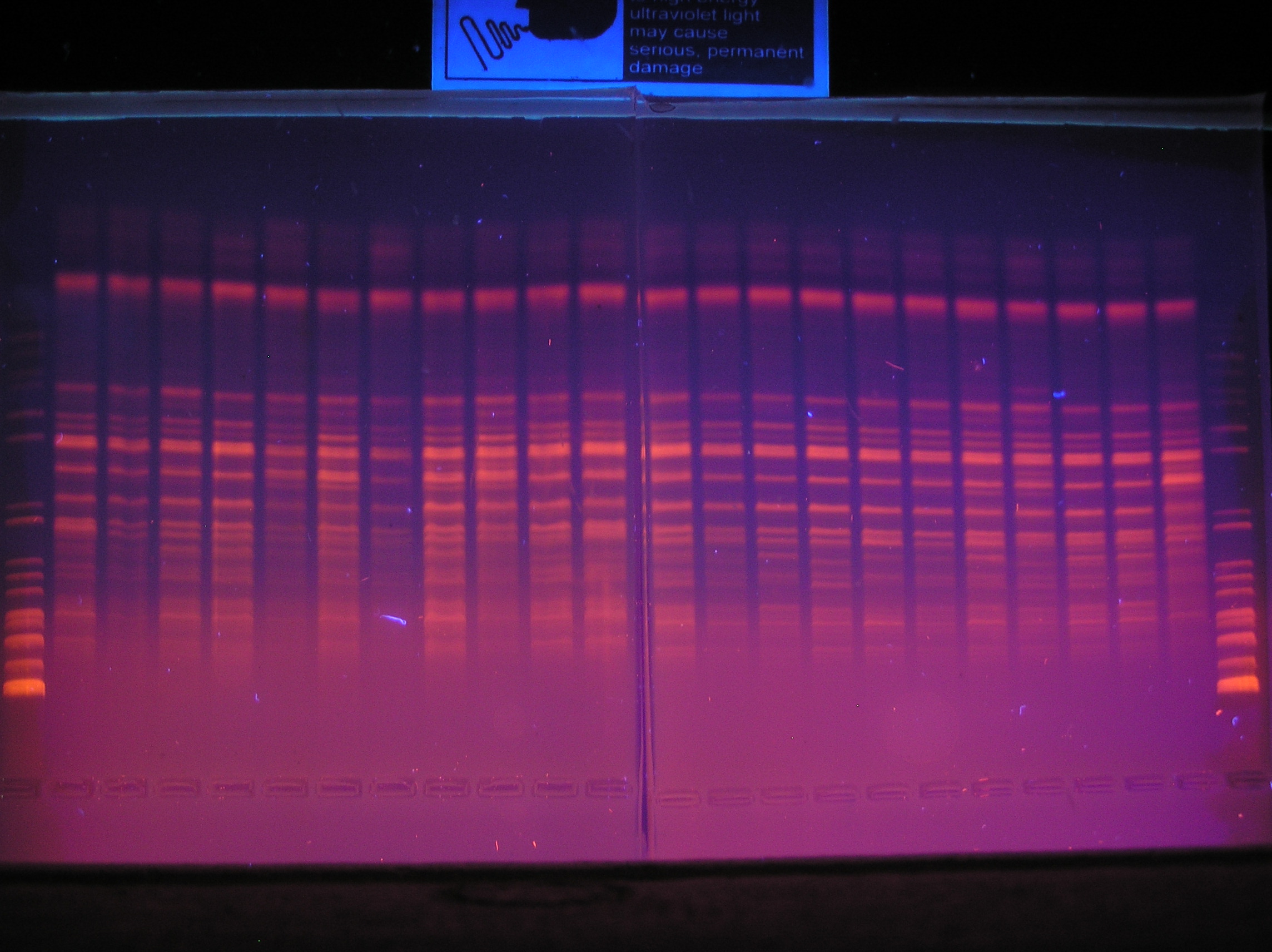

Supplement: Supplemental Information 2 [file peerj-07-6888-s002.zip › iPBS2249/2249_f.JPG]

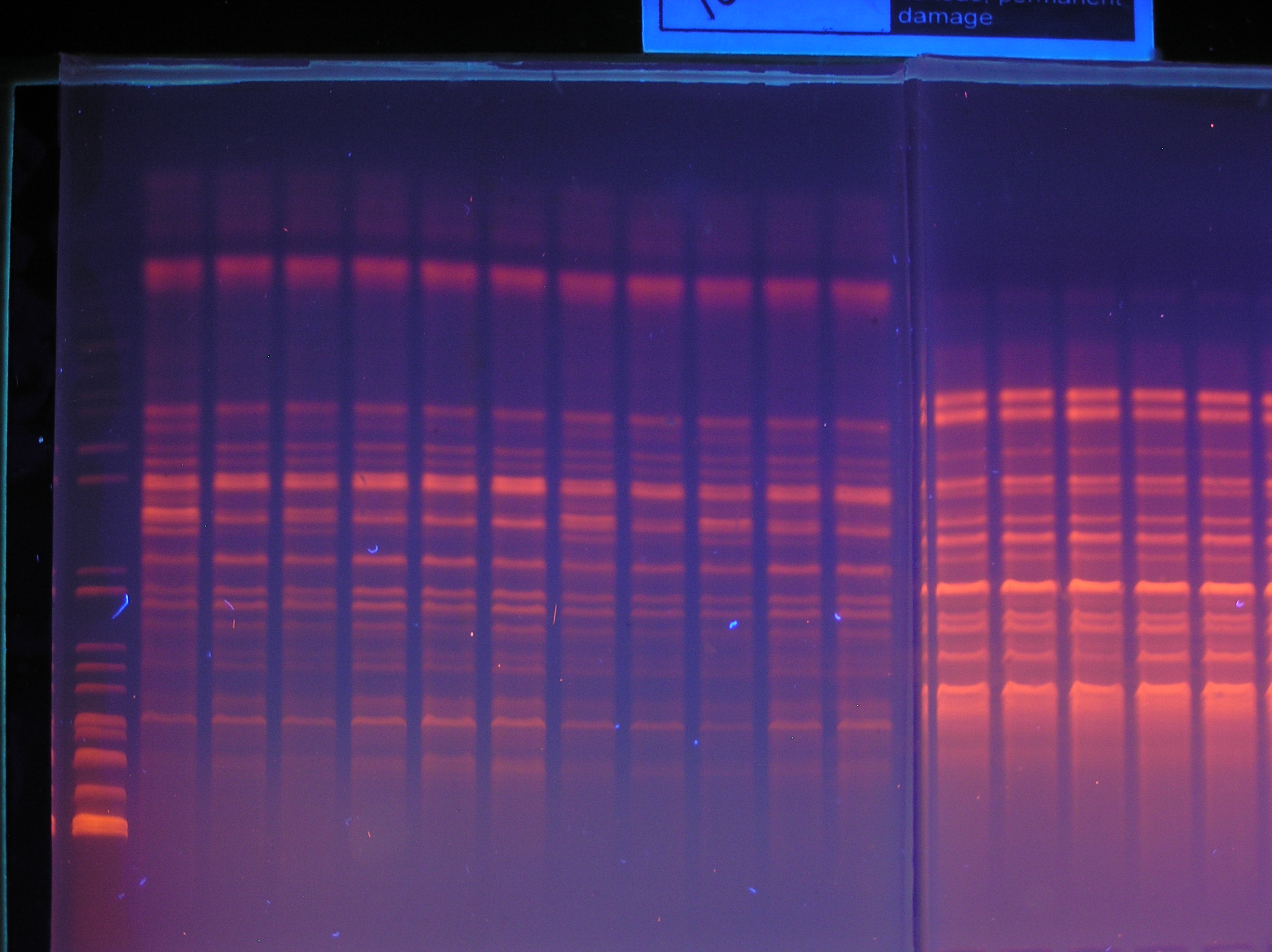

Supplement: Supplemental Information 2 [file peerj-07-6888-s002.zip › iPBS2249/2249_g.JPG]
